# Hospital Saving Association

**Published:** 1924-01

**Authors:** 


### THE HOSPITAL SAVING ASSOCIATION.

WE are glad to learn that the Hospital Saving Association is making steady progress. In addition to the Head Office at 77, Cambridge Terrace, W. 2, several local offices have been opened and the system is working quite satisfactorily. Groups have now been formed in several Government Departments and in the official Institutes, including the Law Courts, where a meeting was held recently. A cordial reception was given to the scheme, and it was decided to ask all the eligible members of the staff to join the Law Courts group. Many hundreds of "H.S.A. Vouchers" have been presented at co-operating hospitals and no case of difficulty arising has been reported to the Association.

### The Financial Aspect.

On the financial side, co-operating hospitals are receiving in respect of each of these cases an interim payment of 7s. 6d. (which compares with an average cost of 7s. 9d. per out-patient at the teaching hospitals) and an interim payment of £2 12s. 6d. weekly towards the cost of maintenance of each in-patient. They have been put to no expense in obtaining this money, the cost of propaganda and administration of the contributory scheme being borne by the Association. These payments are *ad interim*, and a further distribution will be made when the annual accounts are made up. The Association has already secured a regular income of £15,000 for the hospitals, and this sum has been raised by 25,000 members. Up to the end of September over 200 in-patients and over 1,200 out-patients had been treated at co-operating hospitals on the Association's voucher, and no single case of difficulty has been reported. The main obstacle that the Association is finding is in interesting employers.

